# An approach to teaching psychiatry to medical students in the time of Covid-19

**DOI:** 10.1017/ipm.2020.87

**Published:** 2020-07-02

**Authors:** A. Guerandel, N. McCarthy, J. McCarthy, D. Mulligan

**Affiliations:** Department of Psychiatry and Mental Health Research, St. Vincent’s University Hospital and University College Dublin

**Keywords:** Covid-19, medical education, online teaching, student engagement

## Abstract

In this time of Covid-19, life in healthcare has changed immeasurably. It has rapidly been injected with an ‘all hands-on deck’ approach, to facilitate the necessary adaptations required to reduce the spread of the virus and deliver frontline clinical care. Inevitably aspects of these changes have disrupted the delivery of medical education, notably clinical placements have been cancelled and social distancing guidelines prohibit face-to-face teaching. The training of future doctors is an essential part of this effort. Indeed, the emergence of a global health threat has underlined its continued importance. For medical educators and students alike, we have been presented with a challenge. Concurrently, this presents us with an impetus and opportunity for innovation. For some time now, a transformation in medical education has been called for, with an increasing recognition of the need to prepare students for the changing landscape of healthcare systems. This has included a focus on the use of technology-enhanced and self-directed learning. As a team of educators and clinicians in psychiatry, working in the School of Medicine and Medical Sciences (SMMS) in University College Dublin (UCD), we will share how we have responded. We outline the adaptations made to our ‘Psychiatry’ module and consider the influence this may have on its future delivery. These changes were informed by direct student input.

## Introduction

On March 12th 2020, in response to the Covid-19 global pandemic, the Government of Ireland announced the closure of all higher education settings. Additionally, international students were encouraged to return home. For medical students, this marked the abrupt ending of all clinical placements and face-to-face teaching, and a rapid shift to online education. Indeed, worldwide, it seems to have been deemed best practice to remove medical students from clinical environments (Ahmed *et al*. 2020; Rose, 2020). A precedent had been set for this during previous pandemics (Ahmed *et al*. 2020). In University College Dublin (UCD), medical students are taught psychiatry at three junctures; an introduction to psychiatry within the ‘Introduction to Clinical Skills’ module for undergraduate students (and similar teaching for graduate entry students), a stand-alone ‘Psychiatry’ specialty module (in stage 4–5 for undergraduate students, and stage 3–4 for graduate entry students), and as a component of their final ‘Professional Completion’ module. In mid-March, our students were preparing to begin their ‘Psychiatry’ specialty module.

Ordinarily, at this time of year, students rotate through the specialty modules of; Psychiatry, Paediatrics, Obstetrics and Gynaecology, and General Practice. Each module is 6 weeks. They rotate in four groups of approximately sixty students, with each student completing two modules pre-summer, and two post-summer. These are clinically based, and delivered in a hospital or community setting. In these new circumstances, for each specialty, a 2-week online module pre-summer with a (hopeful) plan for a 4-week clinical placement post-summer, was decided upon. This aligned with the broader UCD curriculum and wider university requirements. We will describe the particular approach we have taken to date, in rapidly developing a 2-week online ‘Psychiatry’ module. Despite the time constraints, we did not simply view this as a ‘quick fix’. Instead, keeping the future and advancement of medical education in UCD psychiatry at the forefront of our minds, we saw an opportunity to develop aspects of our teaching delivery.

## The students’ position

**Fig. 1.**
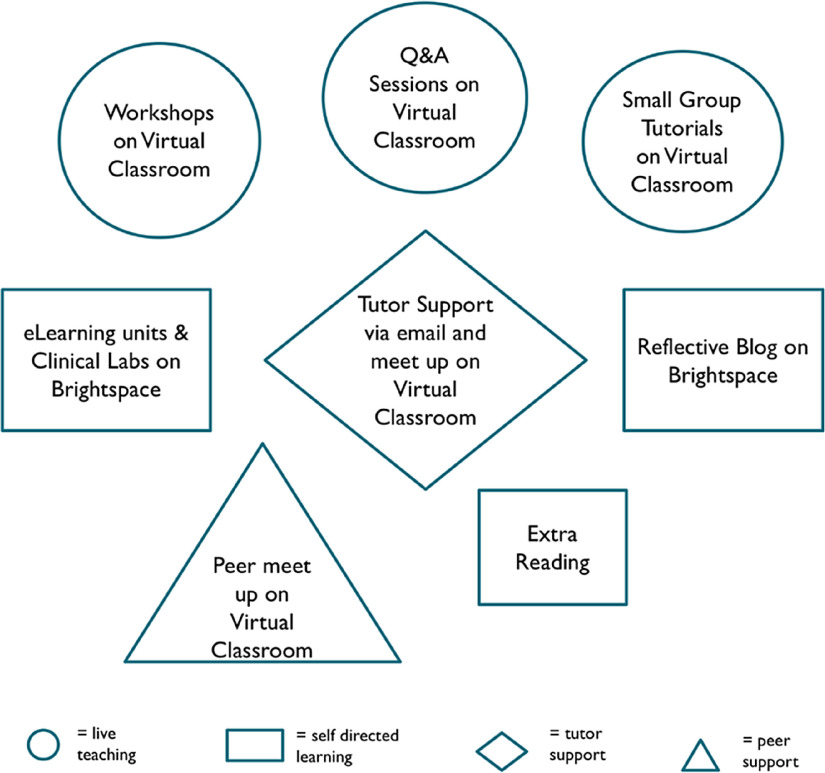
Components of teaching and learning.

Firstly, we considered the students’ position and worked to identify the potential difficulties of being thrust, unexpectedly into an online learning/distance learning environment instead of the usual blended mix of face-to-face teaching supported by online materials. Direct consultations with incoming students were useful for gaining insights. We anticipated that the technological abilities of the students would likely be superior to those of the academics developing the programme. However, inequalities in access to the internet or a suitable device, and competition for internet use within a household may be barriers. The advantages and disadvantages of synchronous (live-teaching and educator-led) and asynchronous learning (without real-time interaction and student-paced) were assessed (Branon & Essex, 2001; Ruiz *et al*. 2006; Eachempati & Ramnarayan, 2020). Notable challenges for synchronous learning included: international students being in different time zones, students joining the frontline effort – for example, as phlebotomists, and the every-day practicalities of students being at home – for example, having to help around the house or care for siblings. Also aware of the unsettling impact of the widespread disruption of ‘normality’, we endeavoured to retain a structured timetable. In addition to live online teaching, we recommended scheduled times for asynchronous learning activities – for example, engaging in e-Learning units. The live teaching was also recorded for students to watch back in their own time. Thus, a blended model of synchronous and asynchronous learning was thoughtfully developed to ensure students were provided with a supportive routine and were able to balance their workload in a personalised, flexible manner. This approach has been recommended by others in the literature (Eachempati & Ramnarayan, 2020). A visual representation of this is displayed in Fig. 1.

Simultaneously, we were sensitive to the psychological shift associated with online learning, and of being a medical student during a pandemic. The impact of social distancing and isolation from family, peers, and the teaching team. Reduced perception of safety with effects on sleep, concentration, motivation, overall wellbeing and academic performance. Uncertainty, precipitating worry, regarding the format of assessments and exams, as well as the future arrangements for the curriculum. Additionally, possible illness and loss. As psychiatrists, we were acutely aware of the mental health effects that can accompany such times (Holmes *et al*. 2020).

## The faculty’s position

Secondly, we considered our teaching faculty and their ability to adapt to the delivery of online teaching, from a technological and psychological perspective. We recognised that using a virtual learning environment (VLE) for all aspects of teaching delivery, may be a daunting prospect for educators with less digital proficiency. Buy-in, commitment, and effective time management would be vital to realise this transformation. We were cognisant that as practising clinicians, all our educators were also experiencing similar upheaval within their clinical work. Additionally, we looked to other clinicians ordinarily linked with our teaching delivery, but not a part of the core team, keeping them updated of developments and seeking out their availability (without overloading their clinical commitments either). This led us to think carefully about our thematic approach to the learning material. We opted to focus on themes with less immediate clinical applicability for students, and (perhaps) comparatively less new demands on the associated teaching clinicians’ time – for example, prioritising forensic psychiatry in the pre-summer module and delaying liaison psychiatry to the post-summer module. In UCD, we are lucky that we have a motivated, skilled, and flexible psychiatry teaching team. Coupled with strong, supportive leadership and organisation, these attributes successfully helped to yield the required changes while continuing the established learning outcomes for the module.

## Module delivery

Thirdly, we considered the mechanisms through which we would deliver the module. At present, medical educators receive frequent correspondence from companies advertising various software packages and even readymade teaching materials. The International Federation of Medical Students Association (IFMSA) (2017) and the Association of Medical Education in Europe (AMEE) (2020) are good sources of evidence-based technology and materials. Specifically, ‘iCollaborative’, via the Association of American Medical Collages (AAMC) (2020), and ‘Pivot MedEd’ are websites designed to support medical educators transition online during Covid-19 (Canadian Medical Education Faculty Development Network, 2020). Nonetheless, we needed to work within what was available to us and our students. In UCD, our VLE is Brightspace (BS). Given the short lead in time, we focused on familiarising ourselves with the ‘Virtual Classroom’ on BS. The team, including our module administrator, attended an instructional webinar to ensure maximum use of its features. Training is considered essential for the success of online education (Eachempati & Ramnarayan, 2020). Fortunately, our team has always been progressive in its efforts to develop online learning supports. In previous iterations of the module we have used digital badges, a bank of videos and e-Learning units on the main themes that we teach, as well as a series of clinical labs and virtual patients. Therefore, building upon this was an opportunity to evolve the module further.

As mentioned above, we used a thematic approach. Ordinarily, this comprises clinical tasks, experiential learning, small group teaching, workshops, and online materials that match weekly themes over 6 weeks. We recognised that it was not possible to cover all themes within just 2 weeks. Therefore, careful choices were made regarding the themes, with an emphasis on developing a solid knowledge base, as the foundation for future learning. As before, the content covered in the live classroom sessions compliments the online learning material. To augment this, references to how students could draw upon the material in preparation for patient interactions pepper the live teaching – for example, case vignettes prompting discussions on how to elicit symptomatology. It is important to note, further teaching is envisioned to take place alongside the (hopeful) clinical placements post-summer. This will cover the remaining themes as well as some revision.

The students used some of the e-Learning units earlier in the curriculum, in their introduction to psychiatry within the ‘Introduction to Clinical Skills’. By directing them to this learning material again, we are enlisting cognitive learning principles used in clinical teaching, such as activating prior learning (Spencer, 2003). To bridge the gap between existing and new information and to contextualise clinical examples, we frequently refer to the material available in the e-Learning units. By opting for scheduled live virtual classes rather than online lectures or podcasts, we aimed to facilitate structure, to help students concentrate on learning at specific times, and to support engagement. The virtual classes allow for asking questions, either verbally or typed. There is a list of attendees available who can ask questions by virtually raising their hands. During live teaching, real clinical cases are discussed and reflection is encouraged. Recordings are made of the live virtual classes, allowing for personal flexibility, revision, and as a ‘back-up’ – for example, if students lose internet connection during class. For some students, having an explicit structure and a set timetable can contribute to preventing feelings of being overwhelmed. (Al-Shorbaji *et al*. 2015).

Online delivery lacks a humane dimension that can potentiate isolation (Liu *et al*. 2016). To achieve engagement, it is also necessary to cultivate a sense of collegiality (Eachempati & Ramnarayan, 2020; Fawns *et al*. 2020). The virtual classroom has cameras and microphones, but to date it has been observed that students in large group workshops do not turn them on, preferring instead to type comments or questions in the text box. Additionally, the virtual classroom has the facility to move students into smaller ‘breakout-groups’ where they can discuss learning points or practice skills. Therefore, the teaching has been organised with regular ‘breakout-moments’ for interactive small group tasks. Here, amongst themselves, we have found that students are more likely to turn on their camera/microphone. Additionally, in the tutor-led small group tutorials, the students are given advance warning that they will be asked to turn their camera/microphone on. Peer social support is encouraged, and the virtual classroom is made available in the timetable for students who wish to participate. Each student is assigned a tutor as a personal point of contact. They correspond directly with them by email, and have a scheduled virtual meeting (with camera/microphone) at the end of the first week. In particular, this support mechanism was born out of direct student input. Lastly, there is an optional online reflective blog and submissions receive constructive feedback from a tutor. Providing diverse channels for interaction allows for varied student preferences.

Historically, student engagement with the module is something we have paid particular attention to. Our continuous assessment includes earning digital badges. Embedded with metadata, they provide an online symbol of accomplishment that can be displayed and shared. These are earned weekly by completing and uploading set tasks. Digital badges are increasingly being used as an innovative way to support learning in medical education (Noyes *et al*. 2020). Linking digital badges to continuous assessment favours a move towards intrinsic motivation to learn. While acknowledging research in this area is in its infancy, a previous study conducted by our teaching team demonstrated their usefulness, particularly in enhancing engagement and motivation (Truszkowska *et al*. 2018). Arguably, these aspects of teaching have never been more needed. In the pandemic conditions of Covid-19, with a palpable absence of the usual external drivers in life, learner motivation needs to shift from extrinsic to intrinsic. Thus, we have retained this system. A digital badge is earned by uploading a self-declaration of attendance, completing a formative online multiple-choice examination (MCQ) and a formative clinical task – for example, watching an online video of a patient interview and describing the mental state examination.

For the structure and delivery of the module, we drew on the principles of the ‘ARCS Model of Motivational Design’ (Keller, 2010). This model prioritises four key motivational factors for the learning environment: attention, relevance, confidence, and satisfaction. To capture attention, we reflected on the current ocular-centric era we live in, acknowledging that students are accustomed to digitally communicating with short texts and imagery (Oxford Reference, 2020). Rather than a didactic teaching style, ample use of diagrams, pictures, and clear messages aims to reduce information overload and stimulate interest. To bring relevance to the material, particularly in the absence of clinical exposure, educators provide lived examples and bring their day-to-day work life into workshops and tutorials. This potentiates the clinical context. The e-Learning units and videos are also useful in creating virtual clinical experiences. Confidence within the students is built through task completion, tutor feedback, and peer interaction and support. Finally, a sense of satisfaction is fostered by earning digital badges.

Clear communication is essential. Reminders about virtual class etiquette follow Medical Council guidelines for online professionalism (Medical Council, 2020). Induction sets the tone with a warm welcome to students and acknowledgement of the circumstances. Determined to rise to the challenge, we drew on the ‘Common Ingroup Identity Model’ as a strategy to foster productive and positive relationships between students and educators (Gaertner & Dovidio, 2000). The basis of this model is an attitude of, ‘we’re all in this together’. In fact, this is akin to the widespread call for social cohesion across the globe during the time of Covid-19. We offered reassurance that, for students and educators in UCD psychiatry, this was indeed the case. Explicit instructions were provided about learning and assessment. Consideration was given to balancing assessment, with 30% pre-summer (enough to drive learning) and 70% during clinical placement post-summer (when most absorbed in learning). Typically, assessment is a worry for students and reassuringly for them, we were able to use elements of our usual online-based assessment, digital badges (5%) and a reflective essay (15%). We will conduct an online MCQ (10%) that will only cover the material that has been taught over the 2-week module. Keeping potential internet inequalities in mind, students are instructed to make contact immediately if there are difficulties. BS allows tracking of attendance and online activity, thus helping us to identify students that need assistance. At this point we have run 1 week of the first iteration of this module, and there have been no difficulties with accessing the online material or any component or modality of teaching delivery.

## Stress management

**Fig. 2.**
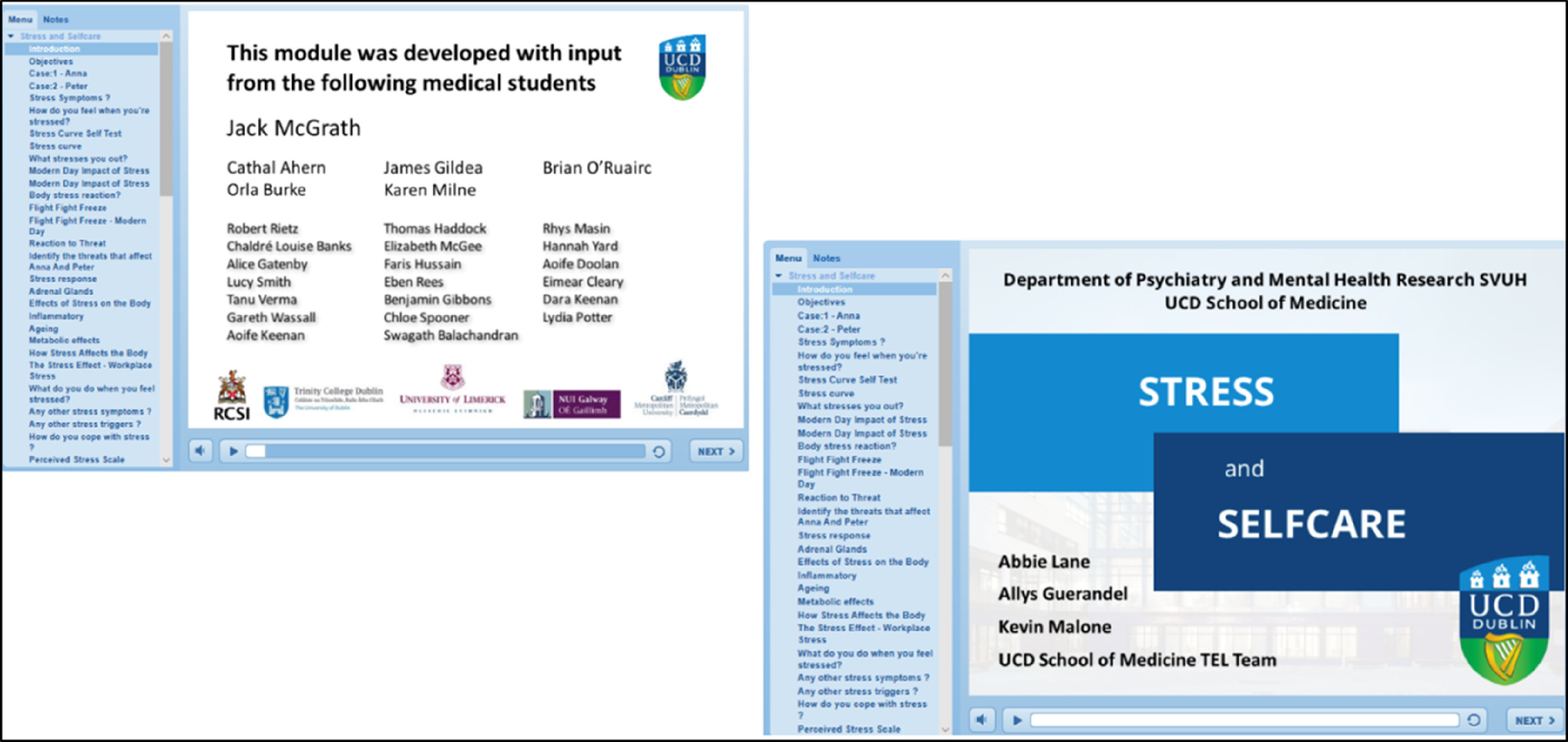
Stress and selfcare eLearning unit.

The Medical Council recognises the need to support students experiencing stress (Medical Council, 2020). In UCD SMMS, we developed an e-Learning unit to help students to recognise, understand, and manage stress (Fig. 2). The unit had direct input from medical students across the national universities. It is embedded in the curriculum and available at each juncture of their psychiatry teaching. Preliminary data indicate its usefulness. Additionally, on this occasion, we have integrated the learning into a workshop titled, ‘Mental Health and Psychological Wellbeing in this Unusual Time’.

Support for faculty is another consideration. In our team, the module administrator has really made his mark, always on hand to manage any technical hiccups, as well as being the key coordinator amongst a suddenly, geographically distant team. Alongside changing academic and clinical practices, the teaching team is also possibly facing additional personal or family health threats, and has friends and colleagues in the frontline of healthcare. Just as for the students, in a pandemic, social connection is vital. The teaching team communicates virtually as a group daily. This communication is quick, easy, supportive and facilitates feedback. Messages are often humorous and accompanied by a few appropriate emojis. Maintaining updates for the associated clinical teams is also important, and similarly for our Objective Structured Clinical Examination (OSCE) examiner team. Regular virtual contact occurs with the wider faculty in SMMS. Furthermore, international virtual collaboration of medical educators is available – for example, a current series of webinars via AMEE.

## Broader context

While uncertainty remains about Covid-19, and its implications for medical education in the short and long term, medical educators have been propelled to embrace the VLE. This embrace has been largely obligated. As with any curriculum development, integration of online education should be done in a planned manner (Kern *et al*. 1998). Of course, in a pandemic, time for such planning is somewhat of a luxury but nonetheless is necessary for success. There are advantages and disadvantages of online learning which require consideration, Table 1. (Al-Shorbaji *et al*. 2015). Evidence exists to support its effectiveness in medical education, but, in particular, when used as an adjunct to traditional teaching methods (Al-Shorbaji *et al*. 2015; Liu *et al*. 2016). The UCD psychiatry teaching team places high value on direct clinical exposure and have been long time advocates of judiciously using online education to compliment this. Indeed, this was the essence of the blended model in place prior to the pandemic. In September, things may have improved enough to have students back on the wards and in the community, but the way this happens may require some modification. Safety will need to be prioritised, with access to, and training for, use of personal protective equipment (Arandjelovic *et al.* 2020). Ultimately, how we respond will be determined in a large part by the practices adopted in our associated clinical sites. Globally, ‘Telemedicine’ is increasingly being used to support continued patient care (Hollander & Carr, 2020). Literature supports technology use for healthcare during public health emergencies (Lurie & Carr, 2018), and it has been suggested that this could also be leveraged for medical education **(**Arandjelovic *et al*. 2020). Although not in place yet in our sites, perhaps ‘Telepsychiatry’ could offer a pathway for sharing consultations with medical students (College of Psychiatrists of Ireland, 2020).

**Table 1.** Advantages and disadvantages of online learning (Al-Shorbaji *et al*. 2015)

	Advantages	Disadvantages
Student Perspective	Accessibility remote settings	Access to internet/device
	Flexibility	Familiarity with technology
	Reduced cost travel/accommodations	Lack of interactions/support with students/educators
	Portable learning materials	Feelings of isolation
	Learning material available for revision	Unable to clarify concepts/have group discussions preferences for face-to-face discussions
	Self paced learning	More time consuming
	Online contact with educators outside scheduled teaching student discussion forums	Difficulty complying with self-guided learning schedule
Educator Perspective	Some cost/time efficient aspects reduces time spent on repetition of learning learning material easily updated/edited reduces equipment/rooms needed	Initial development and investment in e-Learning resources expensive and time consuming
	More standardised learning experience especially if off main campus on clinical placements	Familiarity with technology
	Deliver to large numbers sharing of learning materials across institutions	
	Skill acquisition/practicing prior to real patients fill gaps in clinical exposure	
Teaching Philosophy Perspective	Individualised student-centred student-paced	Teaching certain skills more difficult for example communication skills
	Reduces risk to patients	
	Instantaneous feedback/interactivity aids learning and study habits	

## Conclusion

This are very early days to draw any conclusions from what we are developing, but no doubt there will be lessons to be learnt. At the outset we actively sought direct student input, from an undergraduate and graduate entry viewpoint. Currently, we are gathering widespread informal verbal feedback, which to date has been encouraging and positive. An un-anticipated outcome, has been the recordings of the virtual classes, has facilitated feedback and learning for our tutors who are trainees in medical education. We plan to formalise feedback, using a validated tool, to bring changes forward. We may yet be surprised by our findings. It is very likely that following Covid-19, online teaching will take up a new and exciting position in many courses. As we look towards a future of climate change and an anticipated increase in infectious diseases (Watts *et al*. 2018), medical schools will need to reflect on this and make future investments wisely.
