# An approach to teaching psychiatry to medical students in the time of Covid-19 – Corrigendum

**DOI:** 10.1017/ipm.2021.39

**Published:** 2021-12

**Authors:** A. Guerandel, N. McCarthy, J. McCarthy, D. Mulligan, A. Lane, K. Malone

When originally published, this article didn’t include two of the authors. These authors have now been added and the article has been corrected.

The authors apologise for this oversight.
